# Fine-YOLO: A Simplified X-ray Prohibited Object Detection Network Based on Feature Aggregation and Normalized Wasserstein Distance

**DOI:** 10.3390/s24113588

**Published:** 2024-06-02

**Authors:** Yu-Tong Zhou, Kai-Yang Cao, De Li, Jin-Chun Piao

**Affiliations:** Department of Computer Science and Technology, Yanbian University, Yanji 133002, China

**Keywords:** prohibited object detection, X-ray images, lightweight model, YOLO

## Abstract

X-ray images typically contain complex background information and abundant small objects, posing significant challenges for object detection in security tasks. Most existing object detection methods rely on complex networks and high computational costs, which poses a challenge to implement lightweight models. This article proposes Fine-YOLO to achieve rapid and accurate detection in the security domain. First, a low-parameter feature aggregation (LPFA) structure is designed for the backbone feature network of YOLOv7 to enhance its ability to learn more information with a lighter structure. Second, a high-density feature aggregation (HDFA) structure is proposed to solve the problem of loss of local details and deep location information caused by the necked feature fusion network in YOLOv7-Tiny-SiLU, connecting cross-level features through max-pooling. Third, the Normalized Wasserstein Distance (NWD) method is employed to alleviate the convergence complexity resulting from the extreme sensitivity of bounding box regression to small objects. The proposed Fine-YOLO model is evaluated on the EDS dataset, achieving a detection accuracy of 58.3% with only 16.1 M parameters. In addition, an auxiliary validation is performed on the NEU-DET dataset, the detection accuracy reaches 73.1%. Experimental results show that Fine-YOLO is not only suitable for security, but can also be extended to other inspection areas.

## 1. Introduction

Security screening is an indispensable part of activities such as transportation and accessing sensitive areas. Currently, security screening relies heavily on the pseudo-color images generated from X-ray images by security personnel to determine and identify prohibited objects. However, the increasingly dense traffic networks and large passenger flows have led to a surge in security inspection tasks, which may result in potential instances of missed inspections, endangering the safety of the people and public property. Meanwhile, due to the specificity of the application scenarios, the image produced by the X-ray security channel has its own characteristics, the randomness of the objects to be inspected and the arbitrary placement of the objects in the security process, resulting in a complex background of the X-ray image images. Moreover, the actual application scenario has strict requirements on the detection speed, posing a serious challenge to the detection of prohibited objects. Therefore, it is significant to develop an efficient method to automatically recognize prohibited objects in security X-ray images.

With the rapid development of artificial intelligence technology, convolutional neural network-based vision technologies have found widespread application in image classification and object detection, and has achieved remarkable results. At the same time, an increasing number of scholars have investigated how to utilize computer vision technology to improve the accuracy and speed of detecting prohibited objects in X-ray images. Zhu et al. [1] designed a unique Frequency-aware Dual-stream Transformer (FDTNet) tailored for analyzing X-ray images, introducing a Frequency-Aware Module (FAM) to enhance feature representation using frequency domain information, thus facilitating the accurate detection of prohibited objects. Chen et al. [2] used Discrete Cosine Transform (DCT) to convert RGB domain images into frequency-domain representations, alongside proposing an RGB Frequency Attention Module (RFAM) for comprehensive feature representation integrating RGB and frequency-domain information. Ding et al. [3] introduced FE-DETR, a transformer-based target detection framework that improves anchor-based detectors in foreign object detection through split-attention mechanisms, integration of DCN and CBAM, an MSFE module for feature dispersion processing, and a transformer as a prediction head, along with optimized training strategies to boost detector performance. Wei et al. [4] employed collaborative knowledge distillation and leveraged a teacher model to assist the student model in distillation training, thereby uncovering hard-to-detect prohibited objects in X-ray images. Chang et al. [5] proposed a hard negative sample selection method to generate proposed foreground regions for joint commodity segmentation, aiming to detect prohibited objects in cluttered X-ray baggage images. Hassan et al. [6] utilized incremental learning and a traditional encoder-decoder structure to extract and recognize chaotic, occluded, and overlapping prohibited objects from X-ray images, achieving instance recognition with small-scale training batches. CFPA-Net introduced a cross-layer feature extraction fusion module (CEF) to augment semantic and localization information between low-level and high-level features [7], whereas EAOD-Net incorporated a learnable Gabor revolution layer to enhance the network’s capability to capture edge and contour information about prohibited objects [8].

Although aforementioned methods have been used to address the problems in X-ray images and achieved good detection results, the proposed algorithms are not considered to be applied to realistic scenarios. Considering the limitations of these methods, this study proposes a lightweight detection model that improves both the detection speed and the ability of detecting small prohibited objects. The contributions of this study are as follows:

1. This study proposes a high-density feature aggregation (HDFA) structure for the backbone feature network of YOLOv7, simplifying the network structure and enhancing its ability to capture global object information.
2. A low-parameter feature aggregation (LPFA) structure is proposed for the YOLOv7-Tiny-SiLy neck feature fusion network, which improves the feature integration capability of the lightweight network, resulting in a finer and more comprehensive representation of target features.
3. To avoid the loss of detailed information during feature transmission layer by layer, max-pooling operation is employed for the cross-layer connections. Moreover, the NWD loss function is utilized to enhance the detection of information from small objects given the size constraints of prohibited objects.
4. Experiments conduct on the EDS dataset demonstrate a successful balance between the detection accuracy and speed. Furthermore, the results on the NEU-DET dataset illustrate the robustness of the model and its potential extension to various practical detection domains.

The remainder of this paper is structured as follows. In Section 2, related works on object detection and the X-ray prohibited objects detection are reviewed. The key components of the proposed Fine-YOLO are described in Section 3, such as low-parameter feature aggregation (Section 3.2), high-density feature aggregation and cross-layer connection (Section 3.3), and Normalized Gaussian Wasserstein distance (Section 3.4). Detailed experiments and the analysis are reported in Section 4. Finally, we conclude our work and discuss future work in Section 5 and Section 6, respectively.

## 2. Related Works

### 2.1. Object Detection Algorithms

Object detection constitutes a fundamental task in computer vision, aiming to identify objects of interest within natural images. Existing object detection algorithms can be divided into two categories: two-stage detection algorithms and one-stage detection algorithms. Two-stage detection algorithms, such as Fast R-CNN [9], Faster R-CNN [10], Mask R-CNN [11], and ThunderNet [12], typically generate region proposals prior to making predictions. While effective, these algorithms may exhibit inefficiencies in inference time due to their multi-stage nature. Regarding one-stage object detection algorithms. such as SSD [13], YOLO [14], RetinaNet [15], CenterNet [16], FCOS [17], and DETR [18], directly predict bounding boxes without the need for generating proposals through additional RPNs. This characteristic makes one-stage object detection algorithms particularly suitable for efficient inference on mobile devices. Although DETR has an advantage in accuracy, its complex Transformer structure may require more data and computational resources for training. In contrast, the network architecture of YOLO is designed to be simple, which effectively reduces the amount of computation and model parameters, and thus, is more suitable for the construction of lightweight models.

### 2.2. YOLO Series Object Setection Algorithm

The YOLOv1 algorithm [14] simplifies object detection by dividing images into grids, with each grid responsible for predicting bounding boxes and category probabilities. Although the algorithm achieves fast processing with a single forward pass, it has limited performance in detecting small objects and dense scenes because only one category can be recognized in each grid. To address this problem, YOLOv2 [19] replaces the original GoogLeNet with the more advanced DarkNet-19 network architecture and replaces Dropout with batch normalization, which improves convergence speed and generalization. YOLOv3 [20] introduces the residual structure of ResNet and utilizes Darknet-53 as the base network architecture, resolving gradient explosion issues while increasing network depth. YOLOv4 [21] inherits YOLOv3 and introduces the CSPDarknet53 backbone architecture, which integrates spatial pyramid pooling and path aggregation network (PAN) modules, effectively solving the challenge of varying feature sizes into the fully-connected layer. YOLOv5 [22] retains a similar architecture to YOLOv4; however, it adopts an automatically learned anchor frame based on the training dataset to better adapt to the size distribution of different objects. YOLOv6 [23] brings an innovative Neck network design, featuring a reparameterizable bi-directional fusion Rep-PAN Neck network with enhanced characterization capability. Coupled with a decoupled localization distillation strategy, it boosts the performance of smaller models. YOLOX [24] enhances YOLOv3 by decoupling the prediction branch, separating classification and regression tasks, which speeds up model convergence and improves algorithm generality and versatility.

As the state-of-the-art in the field of image recognition and object detection, YOLOv7 [25] combines extended E-ELAN networks, layer aggregation networks, and composite model scaling strategies. It delivers 5 to 160 frames per second without compromising accuracy in image recognition and object detection. Compared to other YOLO algorithms, YOLOv7 offers the best balance between detection speed and accuracy. By replacing the C3 module in YOLOv5 with the more lightweight C2f module, YOLOv8 [26] achieves a further improvement in network performance. Despite retaining the SPPF module and the design concept of PAN in the YOLOv5 architecture, YOLOv8 simplifies the network structure by removing the convolutional structure of the sampling stage on the PAN-FPN. In addition, YOLOv8 introduces the decoupled-head design with anchor-free detection, which enhances the flexibility and accuracy of the detection algorithm. Notably, YOLOv7 enhances detection accuracy through various improvements, including advancements in the backbone network and feature fusion methods. By incorporating additional feature layers, YOLOv7 is able to capture finer details of the targets, providing a significant advantage in handling complex scenes and diverse objects. Furthermore, YOLOv7 achieves faster detection speeds while maintaining high detection accuracy, making it an exceptionally efficient model for practical applications. Therefore, YOLOv7 is chosen as the baseline model in this study.

### 2.3. X-ray Prohibited Object Detection Datasets

As described in the literature [27,28], X-ray prohibited objects images are significantly different from natural scene images. While natural image datasets like Pascal VOC [29] and MS-COCO [30] are widely known and easily accessible, publicly available datasets of X-ray prohibited objects images are relatively scarce. Accessing natural images is relatively straightforward, as they can be captured using common cameras or smartphones. In contrast, obtaining X-ray images requires specialized equipment. Furthermore, a substantial volume of prohibited objects is essential to assemble the X-ray baggage image dataset utilized for training deep learning-based X-ray prohibited detection models. This necessity significantly compounds the challenge of constructing such a dataset. Moreover, due to privacy policies, many X-ray baggage image datasets cannot be publicly released. To the best of our knowledge, the five current datasets used for X-ray prohibited detection are GDXay [31], SIXray [32], OPIXray [33], HiXray [34], and EDS [35].

GDXray is the first complete dataset that can be used for the detection of prohibited in X-ray security screening, which consists of 19,407 X-ray images. However, this dataset is a greyscale image captured by a single energy X-ray screening machine, and the images scanned by the present X-ray screening machines are all pseudo-colour images, and thus, it does not conform to the present state of research. The SIXray dataset comprises 1,059,231 X-ray images depicting prohibited across six categories: guns, knives, spanners, pliers, scissors, and hammers. However, the dataset faces challenges due to the relatively small number of images containing prohibited, coupled with significant disparities in the quantities of different types of prohibited represented. The OPIXray dataset contains 8885 X-ray images of five different types of objects, namely utility knives, folding knives, utility knives, scissors, and straight knives, with three different levels of occlusion, aimed at investigating occlusion and overlap problems. However, there is a lack of diversity in the dataset as the object objects all belong to the knife category. The HiXray dataset comprises 45,364 X-ray images depicting 102,928 commonly prohibited objects across eight categories. However, among the eight categories, only lighters and rechargeable batteries are prohibited objects.

The EDS dataset is considered to be the first endogenous domain transfer benchmark, crafted specifically for X-ray security screening scenarios. The dataset contains 14,219 X-ray images covering 10 common prohibited categories: beverage bottles, pressure containers, lighters, knives, small electronic devices, power banks, umbrellas, glass bottles, scissors, and laptops. The diversity of these images from screening devices produced by three different manufacturers is suitable for evaluating the proposed Fine-YOLO model.

**Figure 1:**
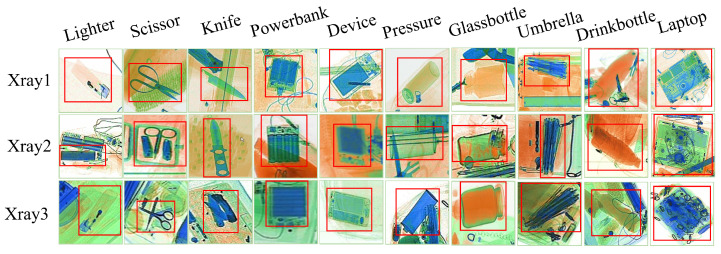
Example of the EDS dataset.

Figure 1 illustrates an example EDS dataset, where Xray1, Xray2 and Xray3 represent images from three different manufacturers of screening machines.

## 3. Methods

### 3.1. Overall Architecture

**Figure 2:**
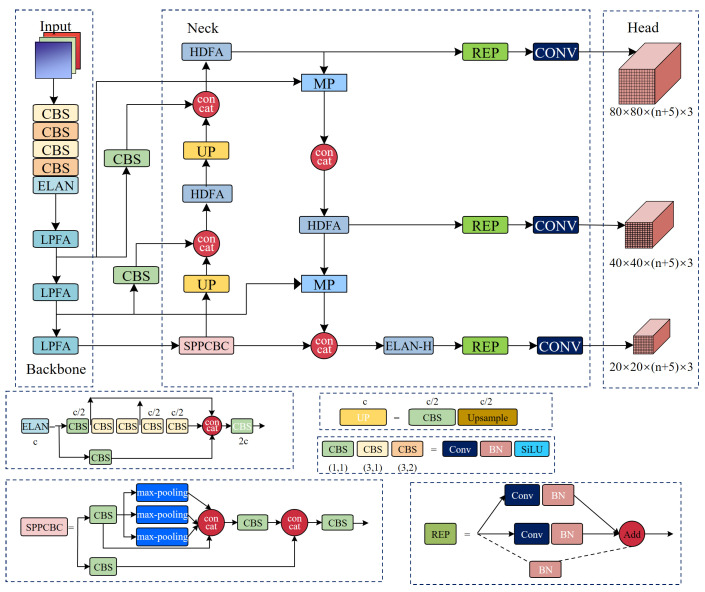
Fine-YOLO network structure diagram.

The framework of the proposed Fine-YOLO is shown in Figure 2. First, the LPFA module with a low number of parameters is proposed for the backbone network of YOLOv7, which is able to better handle the problem of the complex background of X-ray images and the morphological differences of prohibited objects while balancing the performance and computational cost. Then, HDFA module is proposed for the neck feature fusion network of YOLOv7-Tiny-SiLU, enabling the network to capture more detailed feature information and use max-pooling operations for cross-layer connectivity to enhance the prediction effect of the model. Finally, the detection results undergo evaluation using NWD to compute the loss, which in turn guides the optimization of the model.

### 3.2. Low-Parameter Feature Aggregation

**Figure 3:**
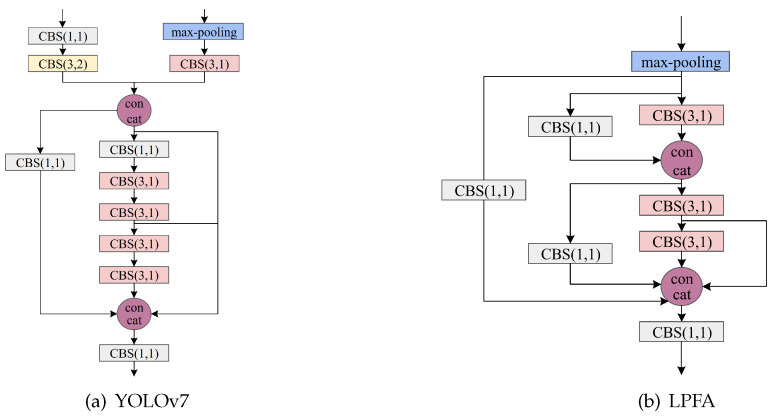
Comparative analysis of improved components within the backbone feature extraction network.

In X-ray images, objects often overlap, making it challenging to detect occluded objects, particularly in areas where the integrity of the occluded objects is compromised. To address this issue, we introduce the LPFA module, designed to enhance the detection accuracy of occluded objects by effectively leveraging global context information within the features. The specific structure of the LPFA module is illustrated in Figure 3.

Compared with YOLOv7, the LPFA module uses the max-pooling operation directly for downsampling, reducing convolutional layers and model complexity while preserving texture details. The resulted features are then processed with 1 × 1 and 3 × 3 convolution kernels, respectively. The 1 × 1 convolution facilitates the interaction of channel information, and the 3 × 3 convolution further extracts more advanced features and semantic insights, expanding the sensory field of the network. After that, the outputs of the different convolutions are concatenated by the concat operation in order for the network to use the different feature information for learning. The two subsequent 3 × 3 and 1 × 1 convolutional kernels function similarly to the above structure with a feature pyramid similar to the feature aggregation structure. Finally, a 1 × 1 convolution is used to recover the dimension and fusion.

### 3.3. High-Density Feature Aggregation

**Figure 4:**
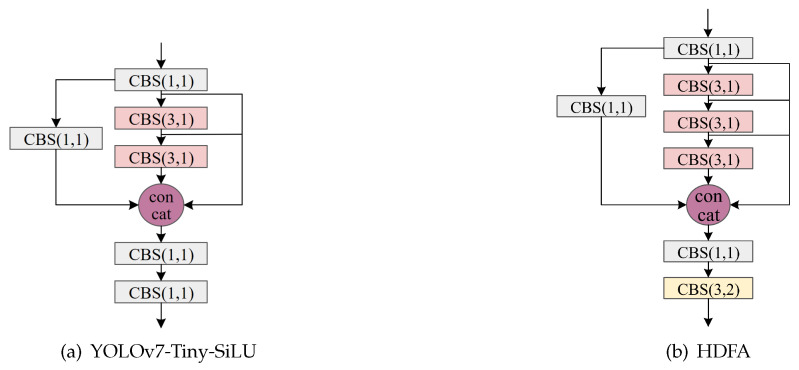
Comparative analysis of improved components within the neck feature fusion network.

Only optimizing the backbone feature extraction network of YOLOv7 obviously cannot achieve a better lightweight effect. Therefore, the HDFA module is proposed in this paper for optimizing the neck feature fusion network of YOLOv7-Tiny-SiLU. YOLOv7-Tiny-SiLU is a compact variant of YOLOv7, which adopts the streamlined network architecture and uses the SiLU activation function on top of the Leaky ReLU. The comparison between the HDFA module and YOLOv7-Tiny-SiLU is shown in Figure 4, where a 3 × 3 convolution is additionally added to the original to improve the feature integration capability of the network. Considering that the neck feature fusion network part is directly connected to the detection head part, if the integration of the features is not strong, it will make the network accuracy decrease and the prediction of the object is not good. The additional 3 × 3 convolution is used because the number of channels in the neck feature fusion network part is small, and even adding a convolution does not result in a very large amount of computation.

**Figure 5:**
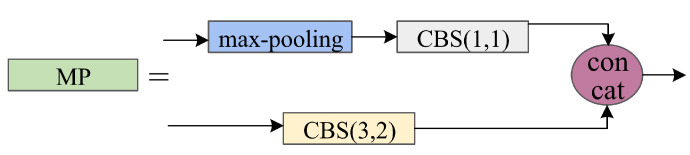
Cross-level connection methods.

To enhance the detection capabilities and facilitate various feature information exchanges within the model, we implement a cross-level connection strategy, which integrated the backbone feature extraction network of YOLOv7 with the neck feature fusion network of YOLOv7-Tiny-SiLU. We address this by introducing a cross-layer network connection that harmonizes the features extracted by both networks. To preserve texture information as much as possible, we employ a max-pooling operation for feature transfer from the backbone feature extraction network to the neck feature fusion network. This strategy enhances feature integration of the entire network, mitigating performance degradation and loss of object prediction. However, due to the large discrepancy between the feature maps referenced by max-pooling and those extracted by multi-layer convolution, directly performing a concat operation can lead to network learning difficulties. Therefore, a convolutional layer is added after max-pooling and the feature maps are adjusted to reduce the discrepancy. The cross-level aconnection method is illustrated in Figure 5.

### 3.4. Normalized Wasserstein Distance

**Figure 6:**
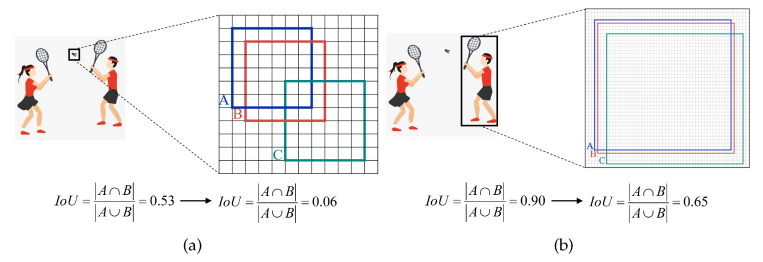
We conduct an analysis of the IoU sensitivity focusing on small- and normal-scale objects. In this analysis, each grid cell represents one pixel. Box A represents the ground truth bounding box, while Boxes B and C illustrate predicted bounding boxes with diagonal biases of one pixel and four pixels, respectively. Specifically, (**a**) pertains sto the detection of micro-scale objects, whereas (**b**) relates to the detection of normal-scale objects.

The IoU (Intersection over Union) metric, commonly employed for object detection, exhibits remarkable sensitivity, particularly in the detection of small prohibited objects. Even slight positional deviations between the predicted and ground-truth bounding boxes can induce substantial fluctuations in the IoU score. As demonstrated in Figure 6, a negligible positional offset could cause the IoU of a prohibited objects small object to plummet from 0.53 to 0.06. However, for objects of regular size, even with the same degree of positional deviation, the IoU only drops from 0.90 to 0.65. The heightened sensitivity of IoU metrics to small prohibited objects leads to significant overlap between positive and negative samples during training, hindering effective network convergence. Additionally, small prohibited objects often occupy minimal space within the image, causing the IoU between the ground truth and predicted bounding box to fall below the minimum threshold, resulting in the absence of positive samples. Hence, there is a pressing need for an alternative evaluation metric to precisely assess small prohibited objects.

(1) Bounding Box Two-Dimensional Gaussian Distribution Modeling

The IoU metric, primarily designed to gauge sample similarity, proves highly sensitive to object size variations, rendering it less suitable for small object detection when objects are prohibited. Hence, we introduce NWD as a novel measure [36]. The Wasserstein distance, non-sensitive to object scales, effectively quantifies distribution similarity with minimal or no overlap. Consequently, it addresses issues related to sample similarity during small object prohibited training and the scarcity of positive samples. Notably, we model the bounding box as a two-dimensional Gaussian distribution:

(1) $$f\left(P|\mu ,\Sigma \right)=\frac{exp(-\frac{1}{2}\left(P-\mu {)}^{T}\sum^{-1}\;\left(P-\mu \right)\right)}{2\pi {\left|\Sigma \right|}^{\frac{1}{2}}}$$

where *P* represents the coordinates $\left(x,y\right)$ and μ and Σ represent the mean vector and covariance matrix of the Gaussian distribution, respectively. In the case of ${\left(P-\mu \right)}^{T}\sum^{-1}\left(P-\mu \right)=\;1$, the ellipse in Equation (1) is the density profile of a two-dimensional Gaussian distribution. Hence, the horizontal bounding box R=(cx,cy,w,h) can be modeled as a two-dimensional Gaussian distribution. The expression is shown in Equation (2):

(2) $$\mu =\left[\begin{array}{c} c_{x}\\ c_{y} \end{array}\right],\Sigma =\left[\begin{array}{cc} \frac{\omega^{2}}{4} & 0\\ 0 & \frac{h^{2}}{4} \end{array}\right]$$

where (cx,cy), *w*, and *h* represent the center coordinates, width, and height, respectively.

(2) Normalized Gaussian Wasserstein Distance

Wasserstein, in Optimal Transport theory, is a common method for calculating the distance between two distributions. This distance measure can facilitate the measurement of the similarity or difference between the two distributions. For two two-dimensional Gaussian distributions $\mu_{a}=N\left(m_{a},\Sigma_{a}\right)$ and $\mu_{b}=N\left(m_{b},\Sigma_{b}\right)$, the Wasserstein distance between $\mu_{a}$ and $\mu_{b}$ is shown in Equation (3):

(3) $$W_{2}^{2}\left(\mu_{a},\mu_{b}\right)=\left|\right|m_{1}-m_{2}{\left|\right|}_{2}^{2}+\left|\right|\Sigma_{a}^{\frac{1}{2}}-\Sigma_{b}^{\frac{1}{2}}{\left|\right|}_{F}^{2}$$

where ${∥\cdot ∥}_{F}$ is the Frobenius norm. Furthermore, the distance between the Gaussian distributions $N_{a}$ and $N_{b}$ modeled by bounding boxes $A=(cx_{b},cy_{b},w_{b},h_{b})$ and $B=\left(cx_{b},cy_{b},w_{b},h_{b}\right)$ can be further simplified to Equation (4):

(4) $$W_{2}^{2}\left(N_{a},N_{b}\right)={\left({\left[cx_{a},cy_{a},\frac{w_{a}}{2},\frac{h_{a}}{2}\right]}^{T},{\left[cx_{b},cy_{b},\frac{w_{b}}{2},\frac{h_{b}}{2}\right]}^{T}\right)}_{2}^{2}$$

Finally, using exponential normalization, the distance can be converted into a value between 0 and 1 to obtain a new metric, Normalized Wasserstein Distance (NWD), as shown in Equation (5):

(5) $$NWD\left(N_{a},N_{b}\right)=exp\left(-\frac{\sqrt{W_{2}^{2}\left(N_{a},N_{b}\right)}}{C}\right)$$

where *C* denotes a constant related to the dataset.

## 4. Experiments

### 4.1. Implementation Details

All experiments in this study are conducted on the Ubuntu 20.04.0 operating system, utilizing an Intel Core i7-12800HX processor, 128 GB of random access memory, and an NVIDIA GeForce RTX 3080 graphics card with 16GB of memory. Python 3.8 is the programming language employed. Image resizing to 640 × 640 is performed, with detector training conducted over 150 epochs. The initial learning rate is set to 0.01, and a batch size of 16 is utilized.

### 4.2. Evaluation Metrics

For evaluation metrics, this paper employs mean Average Precision (mAP), Precision, and Recall to evaluate the detection accuracy of the model. The Average Precision (AP) value represents the average accuracy of object detection within a particular category. The AP value represents the average accuracy of object detection in a particular category. The mAP value represents the average of the AP values across all categories, with an IoU threshold of 0.5. Precision denotes the ratio of correctly detected objects to all detected objects, while recall denotes the ratio of correctly detected objects to positive samples, as illustrated in Equations (6)–(8):

(6) $$Precision=\frac{TP}{TP+FP}$$

(7) $$Recall=\frac{TP}{TP+FN}$$

(8) $$mAP=\frac{\sum_{j=1}^{n}AP\left(j\right)}{n}$$

where TP, FP, and FN denote the numbers of correctly detected objects, false detections, and missed objects, respectively.

In addition, we evaluate the complexity of the model by utilizing the number of parameters and Floating Point Operations (FLOPs), which directly refers to the computational workload. We measure the detection speed of the model, denoted as Frames Per Second (FPS), which represents the number of images processed per second. These metrics are defined in Equations (9) and (10), respectively:

(9) $$Parameters=K^{2}*C_{in}*C_{out}$$

(10) $$FPS=\frac{1}{T_{1}+T_{2}+T_{3}+T_{4}}$$

where *K* denotes the size of the convolution kernel, $C_{in}$ and $C_{out}$ represent the number of input and output channels, respectively, $T_{1}$ represents the preprocessing time before the input image is sent to the neural network, $T_{2}$ represents the the computational inference time of the neural network, $T_{3}$ represents the post-processing time, and $T_{4}$ represents the output time of the result.

### 4.3. Performance of the Fine-YOLO Model

#### 4.3.1. EDS Dataset

This section provides a comprehensive comparison between Fine-YOLO and several classical detection algorithms, as shown in Table 1. The second-stage object detection algorithm, Faster R-CNN, is too slow due to its large number of parameters. SSD has a faster detection speed compared to Faster R-CNN; however, its performance in terms of detection accuracy is very unsatisfactory. RetinaNet is 6.9% higher than SSD in terms of the mAP metrics, although it is nearly twice as slow as SSD in terms of detection speed. The anchor-free family of object detection algorithms, such as CenterNet and FCOS, cannot strike a good balance between detection accuracy and detection speed. In the YOLO series, smaller models such as YOLOv5-S, YOLOX-Nano, and YOLOv8-S, while more lightweight in structure, are not comparable to the Fine-YOLO in terms of detection speed and accuracy. This discrepancy can be attributed to the impact of various factors on model detection speed, including model structure, optimization techniques, hardware efficiency, and implementation details. Despite having fewer parameters, these models may employ more complex network structures or model designs, resulting in greater computational resource requirements and longer inference times. In contrast, Fine-YOLO strikes a balance between model complexity and computational efficiency by optimizing feature extraction, thereby reducing overall execution time. Compared to models such as YOLOv7-Tiny and YOLOv7-Tiny-SiLU, Fine-YOLO provides significant advantages in terms of detection accuracy, speed, and lightweight design for X-ray datasets, despite a slight increase in computational complexity. Moreover, when compared to larger-scale models such as YOLOv5-L, YOLOX-L, YOLOv8-L, and YOLOv7, Fine-YOLO stands out due to its lighter network architecture, faster detection speed, and superior detection accuracy.

**Figure 7:**
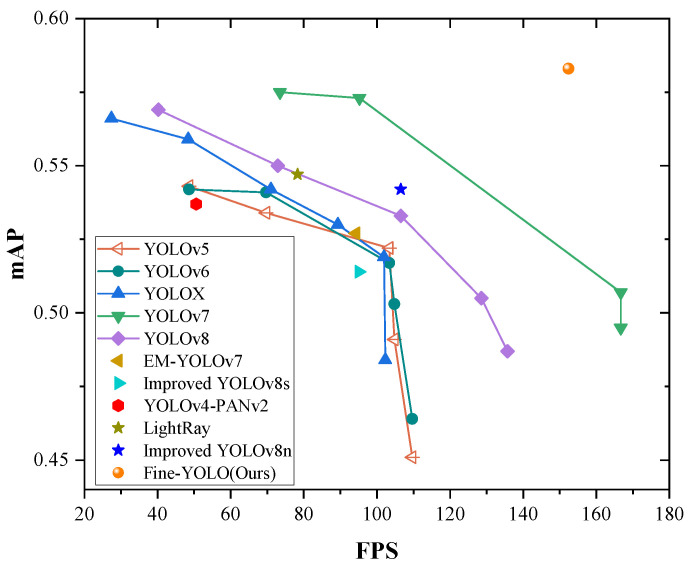
mAP-FPS curves of comparative experiments.

To further validate the efficacy of Fine-YOLO, we compare it with the X-ray prohibited object detection algorithm on the EDS dataset, and the results are presented in Table 2. The methods in the table are all lightweight X-ray prohibited detection methods. The experimental results confirm that our model not only maintains superior detection accuracy, but also reduces model complexity and accelerated detection speed. In summary, Fine-YOLO achieves an excellent balance between lightweight design, high detection accuracy, and rapid detection speed. Its excellent performance in small and large model variants demonstrates that it is an advanced lightweight inspection solution. Figure 7 provides a more intuitive representation of the superiority of our proposed Fine-YOLO algorithm.

#### 4.3.2. NEU-DET Dataset

**Figure 8:**
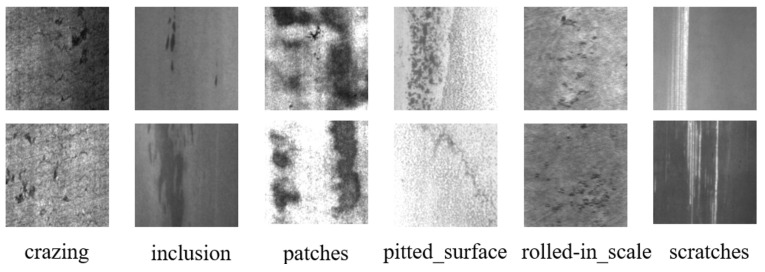
Example of the NEU-DET dataset.

In order to more comprehensively evaluate the performance of Fine-YOLO on different domain datasets and the effectiveness of the proposed improvements on complex background information and small object detection, the NEU-DET dataset is selected for comparative analysis in this paper. The dataset consists of 1800 images containing six different types of surface defects: cracks, inclusions, spots, pitted surfaces, rolled-in scales, and scratches, as shown in Figure 8. In this study, the dataset is split into a 9:1 ratio of training set to test set. The training set is then subdivided into a 9:1 ratio to yield a training subset of 1458 images, a test subset of 180 images, and a validation subset of 162 images.

Experiments are conducted using the NEU-DET dataset, which is then compared with a recently proposed lightweight model for defect detection. The experimental results, presented in Table 3, confirmed the robust performance of our model in the field of small object detection. Notably, our algorithm significantly improves the detection speed while maintaining high accuracy. This makes our model a more convenient solution for object detection applications.

### 4.4. Ablation Study

In this section, we methodically examine the impact of three enhancement techniques on the network model. The experimental findings are detailed in Table 4. Initially, we evaluate the effectiveness of enhancing the backbone feature extraction network using YOLOv7 as the baseline model. Second, the impact of the improvement on the neck feature fusion network is evaluated using YOLOv7-Tiny-SiLU as the baseline model. Subsequently, the effectiveness of the cross-layer connection network is validated. Finally, the NWD loss function is introduced along with a comparison with the performance of YOLOv7. Ten sets of experiments are conducted incorporating different modules, and their performance is evaluated using mAP, Parameters, GFLOPs, and FPS as metrics.

LPFA is an improvement of YOLOv7 backbone feature extraction network, which reduces the number of parameters and computational complexity by the design of low-parameter aggregation module. The proposed method exhibites a remarkable decrease of 67.7% in parameters and 51.6% in computations compared to YOLOv7, while retaining a high detection accuracy. Additionally, our proposed method achieves a 43.9% increase in the detection speed.

HDFA is an improvement of the YOLOv7-Tiny-SiLU neck feature fusion network by designing a high-density aggregation module that efficiently integrates features at different scales, thus improving the performance of object detection and the dependence on background information. Despite the increased parameters and computational resources of our proposed method compared to YOLOv7-Tiny-SiLU, the detection accuracy is improved by 2.2%.

By connecting the LPFA and HDFA modules through a max-pooling operation, this network architecture offers significant advantages over YOLOv7. Notably, Fine-YOLO achieves a 56.7% reduction in the number of parameters, 44.9% decrease in computational load, and 60.1% increase in detection speed. This facilitates the improvement of the detection speed while maintaining high detection accuracy, rendering it easier for the actual application of the model. In the pursuit of lightweight design, we observes a potential impact on the detection accuracy of the model when verifying the above method.

**Figure 9:**
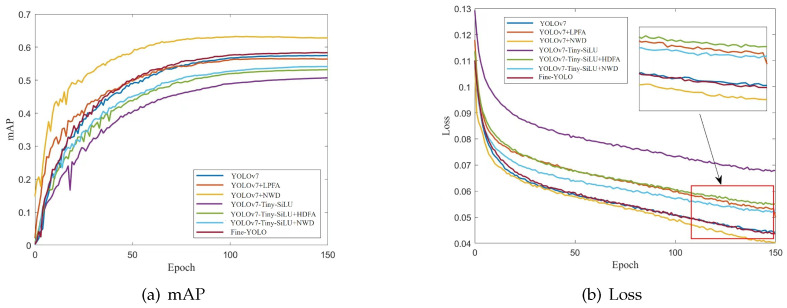
Ablation experiment results of improved methods.

To address this issue, we introduce the NWD loss function in this article, aimed at striking a balance between detection accuracy and speed. This enhancement yields a 1.0% improvement in detection accuracy compared to YOLOv7. The introduction of the NWD loss function effectively solves the problem of bounding box regression mismatch when detecting small objects and further improves the detection accuracy while maintaining a lightweight design. Figure 9 illustrates the results of the ablation experiments for the improved methods.

### 4.5. Visualization of the Detection Result

**Figure 10:**
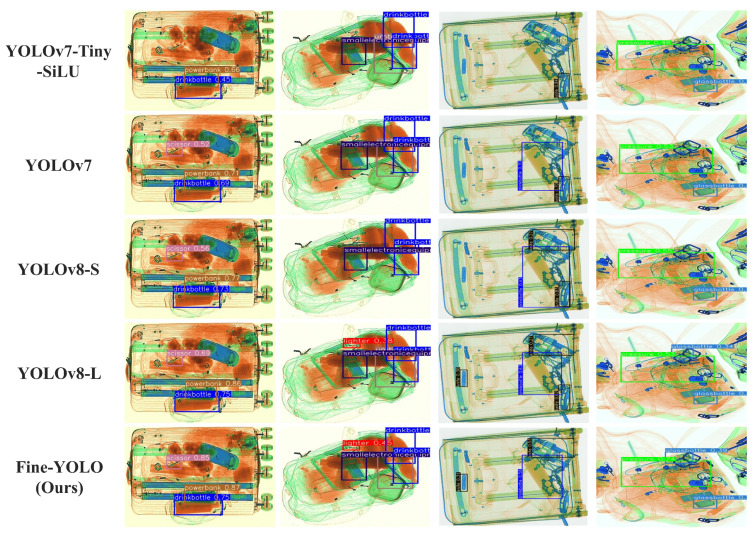
Comparison of the prediction effect of different models on the EDS dataset.

Figure 10 shows the comparison results of YOLOv7-Tiny-SiLU, YOLOv7, YOLOv8-S, YOLOv8-L, and Fine-YOLO. It is visualized from the figure that Fine-YOLO is able to accurately identify small prohibited objects such as scissors, knives, lighters, etc., and has a higher confidence level for slightly larger objects such as laptops and bottles.

## 5. Discussion

According to the above experimental results and analyses, it can be proved that Fine-YOLO has obvious advantages in X-ray security detection and can balance well the detection accuracy and detection speed. There are three main innovations. (1) A low-parameter feature aggregation module is proposed for the backbone network of YOLOv7. This design enables the model to capture global information efficiently while maintaining a lightweight structure. (2) A high-density aggregation feature model is introduced for the feature extraction network of YOLOv7-Tiny-SiLU. This enhancement allows the model to maintain its lightweight nature while simultaneously improving detection accuracy. (3) Considering the different structures of YOLOv7 and YOLOv7-Tiny-SiLU, a cross-layer connection is established using max-pooling operations. In light of the above discussion, the superiority of Fine-YOLO is mainly manifested in the following aspects: (1) Two different feature fusion modules are designed according to the characteristics of YOLOv7 and YOLOv7-Tiny-SiLU models, which achieves a good balance between detection speed and accuracy. (2) For different network structures, max-pooling operation is employed for cross-layer connection, thereby minimizing the loss of feature information. (3) The application of the NWD loss function further enhances the detection accuracy of the model without sacrificing the lightweight design advantage of the model, particularly for small prohibited objects. Notably, the auxiliary validation conducted on the NEU-DET dataset confirms the excellent detection performance of Fine-YOLO and shows the potential to be extended to other object detection domains.

## 6. Conclusions

This study presents the Fine-YOLO model for detecting prohibited objects in X-ray images. Despite the challenges of complex background and small size of prohibited, the Fine-YOLO model proposed in this paper shows excellent detection performance. The major contributions are as follows: (1) The LPFA module is proposed for the YOLOv7 backbone feature extraction network to extract the multi-scale global context information, which contributes to the detection of occluded objects; (2) The proposed HDFA module enables the neck feature fusion network of YOLOv7-Tiny-SiLU to capture detailed object information while possessing a lower number of parameters; (3) The features of the backbone feature extraction network and the neck feature fusion network are effectively merged by max-pooling operation; (4) The model is optimized using the NWD loss function to enhance detection accuracy for small objects. Extensive experimentation and visualization analyses demonstrate that Fine-YOLO precisely identifies and classifies prohibited objects, achieving faster detection speeds and a lighter network structure. Experiments performed on the EDS dataset and auxiliary validation on the NEU-DET dataset demonstrate the excellent detection performance of Fine-YOLO. However, the unique characteristics of the security screening dataset, such as frequent stacked placements in X-ray images, pose challenges that may hinder overall algorithm performance.

In future research, we aim to utilize reinforcement learning technique to further improve the detection accuracy of prohibited objects through an iterative trial-and-error and feedback process. In addition, we plan to explore optimization techniques such as knowledge distillation and model pruning to improve the efficiency and applicability of the model. Furthermore, we intend to integrate LPFA and HDFA modules into other YOLO algorithms to refine their architectural design and ensure improved detection capabilities.

## Figures and Tables

**Table 1:** Experimental results of different object detection algorithms on the EDS dataset. It is worth noting that in this study, baseline model values are underlined, whereas optimal values are in bold.

Method	Precision	Recall	mAP	Params.	GFLOPs	FPS
Faster R-CNN [10]	0.603	0.451	0.491	136.9 M	369.9	16.0
SSD [13]	0.621	0.432	0.405	24.8 M	61.7	89.4
RetinaNet [15]	0.653	0.442	0.474	36.5 M	148.2	46.3
CenterNet [16]	0.614	0.461	0.489	32.7 M	70.2	19.4
FCOS [17]	0.675	0.542	0.559	32.1 M	161.5	42.5
YOLOv5-N [22]	0.606	0.441	0.451	1.8 M	**2.6**	156.3
YOLOv5-S [22]	0.624	0.477	0.491	7.1 M	15.2	133.3
YOLOv5-M [22]	0.675	0.488	0.522	20.9 M	26.8	111.1
YOLOv5-L [22]	0.704	0.498	0.534	46.2 M	73.8	77.5
YOLOv5-X [22]	0.714	0.516	0.543	86.3 M	155.7	47.2
YOLOX-Nano [24]	0.618	0.413	0.484	**0.9 M**	2.8	102.3
YOLOX-Tiny [24]	0.646	0.448	0.519	5.0 M	4.3	101.9
YOLOX-S [24]	0.651	0.454	0.530	8.9 M	16.0	89.3
YOLOX-M [24]	0.663	0.480	0.542	25.3 M	48.3	71.0
YOLOX-L [24]	0.672	0.496	0.559	54.1 M	108.4	48.4
YOLOX-X [24]	0.675	0.504	0.566	99.0 M	204.8	27.4
YOLOv6-N [23]	0.614	0.496	0.464	4.3 M	11.1	109.7
YOLOv6-T [23]	0.649	0.511	0.503	9.7 M	24.8	104.8
YOLOv6-S [23]	0.660	0.520	0.517	17.2 M	44.1	103.4
YOLOv6-M [23]	0.673	0.534	0.541	34.2 M	82.0	69.7
YOLOv6-L [23]	0.679	0.533	0.542	58.5 M	143.8	48.6
YOLOv7-Tiny [25]	0.597	0.489	0.495	6.0 M	13.3	**166.7**
YOLOv7-Tiny-SiLU [25]	0.643	0.481	0.507	6.0 M	13.1	**166.7**
YOLOv7 [25]	**0.721**	0.532	0.573	37.2 M	103.3	95.2
YOLOv7-X [25]	0.701	0.546	0.575	70.9 M	189.1	73.5
YOLOv8-N [26]	0.562	0.502	0.487	3.0 M	8.2	135.7
YOLOv8-S [25]	0.591	0.498	0.505	11.1 M	28.7	128.6
YOLOv8-M [25]	0.637	0.516	0.533	25.9 M	79.1	106.5
YOLOv8-L [25]	0.659	0.526	0.550	43.6 M	165.4	72.9
YOLOv8-X [25]	0.667	**0.544**	0.569	68.2 M	258.1	40.2
Fine-YOLO (Ours)	0.719	0.536	**0.583**	16.1 M	56.9	152.4

**Table 2:** Performance comparison of Fine-YOLO with different lightweight X-ray inspection models, where bold indicates the optimal value for each metric.

Method	Precision	Recall	mAP	Params.	GFLOPs	FPS
LightRay [37]	0.667	0.519	0.547	19.0 M	52.5	78.3
YOLOv4-PANv2 [38]	0.665	0.502	0.537	15.2 M	38.5	50.6
EM-YOLOv7 [39]	0.640	0.512	0.527	37.2 M	103.3	94.3
Improved YOLOv8s [40]	0.593	0.507	0.514	**11.5 M**	**30.6**	95.0
Improved YOLOv8n [41]	0.633	0.491	0.542	25.8 M	79.1	106.5
Fine-YOLO (Ours)	**0.719**	**0.536**	**0.583**	16.1 M	56.9	**152.4**

**Table 3:** Performance comparison between Fine-YOLO and different advanced lightweight steel defect detection models, where bold indicates the optimal value for each metric.

Method	Precision	Recall	mAP	Params.	GFLOPs	FPS
RDD-YOLO [42]	0.561	0.634	0.638	8.9 M	13.9	130.4
DCAM-Net [43]	0.640	**0.735**	0.725	30.5 M	73.0	100.2
Regularized YOLO [44]	0.638	0.675	0.703	**3.0 M**	**8.3**	131.4
Improved YOLOX [45]	0.652	0.697	0.723	7.2 M	20.7	100.0
Fine-YOLO (Ours)	**0.698**	0.722	**0.731**	16.1 M	56.9	**161.3**

**Table 4:** Ablation experiments on the EDS dataset.

Model	LPFA	HDFA	NWD	mAP	Params.	GFLOPs	FPS
YOLOv7				0.573	37.2 M	103.3	95.2
	√			0.564	12.0 M	50.0	137.0
			√	0.627	37.2 M	103.3	95.2
	√		√	0.574	12.0 M	50.0	137.0
YOLOv7-Tiny-SiLU				0.507	6.0 M	13.1	166.7
		√		0.529	8.5 M	17.3	161.3
			√	0.541	6.0 M	13.1	166.7
		√	√	0.552	8.5 M	17.3	161.3
Fine-YOLO	√	√		0.567	16.1 M	56.9	152.4
	√	√	√	0.583	16.1 M	56.9	152.4

## Data Availability

The data that support the findings of this study are available on request from the corresponding author.

## Abbreviations

The following abbreviations are used in this manuscript:

YOLO	You Only Look Once
LPFA	low-parameter feature aggregation
HPFA	high-density feature aggregation
NWD	Normalized Wasserstein Distance
mAP	mean Average Precision
AP	Average Precision
Params.	parameters
FLOPs	Floating Point Operations
FPS	Frames Per Second
IoU	Intersection over Union
